# Transient left ventricular dysfunction in Churg Strauss syndrome: a case report

**DOI:** 10.4076/1757-1626-2-6564

**Published:** 2009-07-16

**Authors:** Ioannis Vlahodimitris, Maria Christina Kyrtsonis, Nikolaos Lionakis, Vassilios Votteas, Ioannis Moyssakis

**Affiliations:** 1 Cardiac Department, Laiko General Hospital of Athens17 Agiou Thomas St. GR-15727 Goudi, AthensGreece; 2 Haematology Department Laiko General Hospital of Athens17 Agiou Thomas St. GR-15727 Goudi, AthensGreece

## Abstract

A 42 year old woman was admitted to our hospital for investigation of eosinophilia. There were no findings from the physical examination of the lungs and heart. The echocardiography showed a segmental hypokinesia of the interventricular septum and the apex causing left ventricular dysfunction with an ejection fraction 45% and mild pericardial effusion. Cardiac magnetic resonance was performed, for detection of lesions associated with the underline disease, using electrocardiogram-triggered T2-weighted and T1-weighted multislice spin-echo images (before and after an intravenous bolus of gadolinium).

The analysis of T2-weighted images revealed increased signal on the mid part of interventricular septum, suggesting myocardial oedema. In the delayed-enhanced images, areas of late phase gadolinium enhancement (indicative of fibrosis) were identified in the mid part of interventricular septum. Methylprednisolone therapy was started. The patient had follow-up echocardiographic examination every month and on sixth month improvement of left ventricular dysfunction was shown with an ejection fraction 55%.

In conclusion our case is a typical Churg Strauss Syndrome with characteristic myocardial involvement which improved after corticosteroid treatment. The cardiac magnetic resonance has significant role for early and accurate detection and differentiation of myocardial damage even in preserved cardiac wall motion and cavity size.

## Introduction

Churg Strauss syndrome (CSS) is a rare necrotizing systemic vasculitis [1]. In the initial phase asthma and rhinitis are the most common manifestations of the syndrome. The next phase of the disease is the peripheral and tissue eosinophilia and finally comes the vasculitis of different organs with life threatening results [2,3]. Cardiac involvement is frequent in patients with CSS and predicts the final prognosis [4,5].

## Case presentation

**Figure 1.**
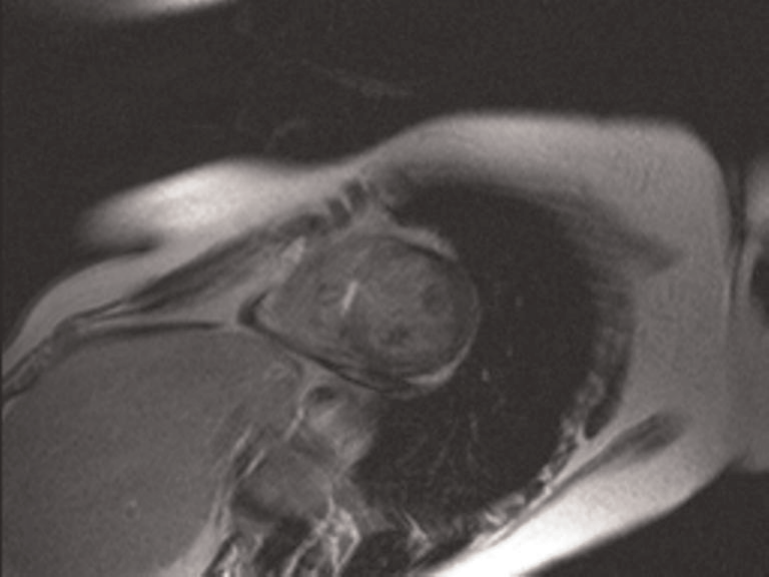
Short axis delayed-enhanced image of left ventricle after injection of gadolinium, indicative of inflammation in the interventricular septum.

**Figure 2.**
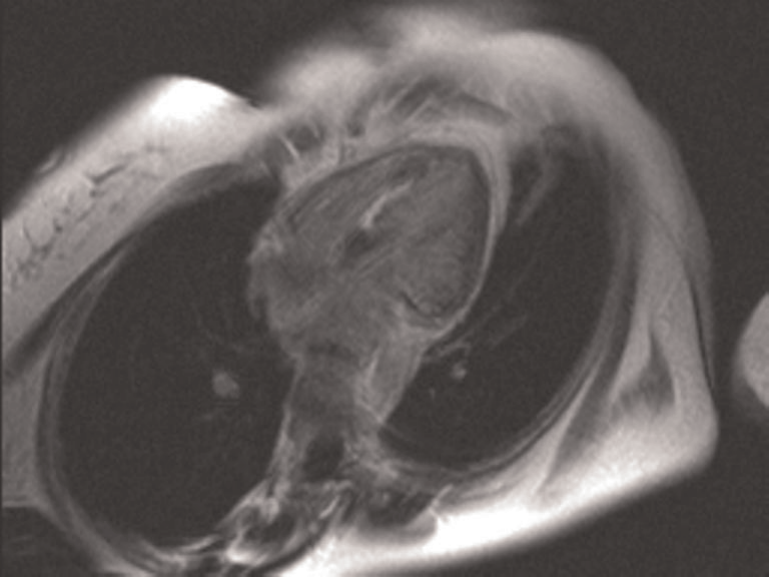
Four chamber delayed-enhanced image of left ventricle after injection of gadolinium, indicative of inflammation in the interventricular septum.

A 42 year old Greek woman was admitted to our hospital for investigation of eosinophilia. On admission, the white blood cells were 24, 2 K/μl with 62.8% the eosinophils. She had suffered from bronchial asthma and recurrent sinusitis for the last 4 and 6 years respectively. There were no findings from the physical examination of the lungs and heart. The blood pressure was 110/70 mm/Hg whereas the electrocardiogram and chest x-ray were normal. The echocardiography showed a segmental hypokinesia of the interventricular septum and the apex causing left ventricular dysfunction with an ejection fraction (EF) 45% and mild pericardial effusion. Valvular disease or thrombus formations were excluded. Cardiac enzymes as serum creatine kinase-MB and troponin T were in normal levels. Cardiac magnetic resonance (CMR) was performed, for detection of lesions associated with the underline disease, using electrocardiogram-triggered T2-weighted and T1-weighted multislice spin-echo images (before and after an intravenous bolus of 0.1 mmol/kg gadolinium). The analysis of T2-weighted images revealed increased signal on the mid part of interventricular septum, suggesting myocardial oedema. In the delayed-enhanced images, areas of late phase gadolinium enhancement (indicative of fibrosis) were identified in the mid part of interventricular septum. The EF of the left ventricle (LV) was 50% whereas a mild pericardial effusion was also noted (Figure 1 and Figure 2). Other causes of eosinophilia were excluded by bone marrow biopsy. Therapy was started with methylprednisolone 32 mg per day. The patient had follow-up echocardiographic examination every month and on sixth month improvement of left ventricular dysfunction was shown with an EF 55%.

## Discussion

CSS is a rare necrotizing vasculitis of small blood vessels (arteries and veins), with eosinophilic infiltration of them [6]. An estimation about the frequency of this particular vasculitis in the population is 2.4 - 6.8 cases in 1.000.000 patient per year [7]. Late onset of asthma, nasal polypus and sinusitis are the first manifestations of the syndrome in the early phase [2,7,8]. The second clinical phase is tissue eosinophilia and pulmonary infiltrations, while the last phase of the syndrome is vasculitis of many organs such as skin, gastrointestinal, lung, heart and peripheral nervous system [3]. This particular phase is life threatening, especially when there is heart and gastrointestinal involvement [3,7]. The syndrome affects both males and females and the mean age of presentation is usually 40 years old [9]. The asthma and its corticosteroid therapy, sometimes cover, for a period of time, the manifestations of vasculitis [7,9]. The onset of the vasculitic phase usually reduce the severity of the asthma, but its presence is longstanding in approximately 80% of the patients [7,10]. The American College of Rheumatology developed in 1990 the diagnostic criteria for CSS. Four of the 6 criteria make the diagnosis almost accurate. The criteria include asthma, eosinophilia (more than 10% of the white blood cell count), pulmonary infiltrates, mononeuropathy or polyneuropathy, paranasal sinus abnormality and final extravascular eosinophil infiltration on biopsy [1,6,10]. Usually the asthma is followed by peripheral and tissue eosinophilia with final result the transient pulmonary infiltrations. The patients have findings in their chest x-ray ranging from 62-77% [9]. The combination of asthma and eosinophilia helps to distinguish this particular syndrome from other vasculitis like Wegener and microscopic polyangiitis and from other blood disease like eosinophilic leukemias and hypereosinophilic syndrome in which there is absence of asthma [3,7]. The final stage is the vasculitis and it involves many organs like skin, lungs, nervous system, gastrointestinal tract, kidneys and heart. Common manifestations are purpura, peripheral neuropathy, segmental necrotising glomerulonephritis, pleural effusion, gastrointestinal perforation and haemorrhage. The last usually predicts poor prognosis [6,7,9]. It should be noted also that CSS is ANCA (antineutrophil cytoplasmic antibodies) associated vasculitis. Approximately 70-80% of the patients with CSS are ANCA positive (especially with the perinuclear type) [6,10]. The presence of ANCA correlates with renal involvement, while the absence correlates with cardiac manifestations [6].

Cardiac involvement is common in CSS and it has important prognostic role. It occurs in 40-47% of the cases [4,5]. More often causes epicardial coronary vasculitis, valvular disease, congestive heart failure due to eosinophilic myocarditis, pericarditis and pericardial effusion [2,6,10,11]. Myocardial infraction is the result of the epicardial coronary vasculitis [2], while the pericardial effusion might cause congestive symptoms and rarely cardiac tamponade [6,9]. The fluid is exudate with marked eosinophilia and low levels of glucose. It has also been reported the case of atraumatic intra- pericardial thrombosis [5]. The early diagnosis of the cardiac involvement has major importance because the development of heart failure makes the prognosis poor [12]. The diagnostic tools are echocardiography and cardiac magnetic resonance (CMR). The last has important role because findings like myocardial fibrosis with subendocardial location, oedema and hyperemia have early appearance and echocardiography cannot detect them [4].

Corticosteroids are the first line therapy of CSS causing remission and survival improvement. Prednisone 1mg/kg day is the recommended dose for 1 month with gradual taper up to 1 year [7,9]. When the recurrences are frequent, or there is a serious form of necrotizing vasculitis in an organ like the gastrointestinal tract or the heart, then the usage of cyclophosphamide is recommended. The combination therapy controls the relapses of disease but has no advantage in survival. In addition this particular therapy has serious side effects with most important the risk of urological malignancies. This results from the accumulation of the metabolite acrolein in the bladder [7].

In conclusion our case is a typical CSS syndrome with characteristic myocardial involvement which improved after corticosteroid treatment. Since cardiac involvement is a leading cause of mortality it is crucial to detect cardiac involvement as early as possible [12]. The CMR has significant role for early and accurate detection and differentiation of myocardial damage even in preserved cardiac wall motion and cavity size.

## Abbreviations

ANCA: Antineutrophil cytoplasmic antibodies
CCS: Churg Strauss Syndrome
CMR: Cardiac magnetic resonance
EF: Ejection fraction
LV: Left ventricle
